# Pediatric Red Ear Syndrome Misdiagnosed as Relapsing Polychondritis: A Case Report and Review of Literature

**DOI:** 10.1155/crpe/6464822

**Published:** 2025-01-20

**Authors:** Nicolas Sandakly, Georgio El Koubayati, Jeannette Sarkis, Fady Haddad

**Affiliations:** ^1^Faculty of Medical Sciences, Lebanese University, Hadath Campus, Lebanon; ^2^Department of Internal Medicine, Lebanese Hospital Geitaoui University Medical Center, Beirut, Lebanon

## Abstract

Red ear syndrome (RES) is a rare clinical entity presenting with paroxysmal erythema of one or both ears associated with a burning sensation or earache. The onset of symptoms could be either spontaneous or triggered by touch, stress, coughing, sneezing, neck movements, chewing, and combing hair. While most cases are usually described in adults, it can rarely present in children. This article reports a case of pediatric RES, first misdiagnosed as relapsing polychondritis. The patient's condition improved after introduction of ibuprofen, isoptin, and amitriptyline, with no new attacks reported after a 1 year follow up.

## 1. Introduction

RES is a rare condition characterized by episodic erythema and pain felt over the pinna of the ear, that can radiate toward the cheek and mandible [1]. Pain is often described as a burning sensation, though less commonly; it has been described as dull or stabbing. The erythema invariably parallels the pain and persists as long as the burning pain does [1]. While initially thought to cause only unilateral attacks, bilateral attacks have also been reported [2]. Triggering factors can be touch, exertion, heat or cold, stress, neck movements, sneezing, coughing, chewing, and/or brushing of hair, but the symptoms can also start spontaneously without any precipitating conditions [3]. Its prevalence and incidence remain unknown, with fewer than 100 cases reported since Lance first described it in 1995. The median age of onset of RES is 44 years. We hereby report a rare case of pediatric RES, first mistaken for relapsing polychondritis (RP).

## 2. Case Presentation

A 14 year-old-male patient presented to the Lebanese Hospital Geitaoui University Medical Center (LHG-UMC) with severe pain and erythema of the left ear and unilateral conjunctival injection. The patient's symptoms began 5 years ago prior to admission, characterized by a sudden onset of pain over the pinna of the ear radiating toward the cheek. The pain was described as a burning sensation, occasionally a dull ache lasting from 10 to 20 min to two hours, although sometimes, the attacks lasted over five to six hours and were associated with constant pain. These episodes occurred in clusters that lasted up to 4 months. During the clusters, the episodes occurred up to five times per day. The attacks were unilateral and non-side-locked. The pain was associated with warmth of the skin. No precipitating factors were identified by the patient or his parents.

Initially, the patient was treated with corticosteroids and pregabalin for a presumed diagnosis of RP which resulted in partial resolution of the symptoms and a decrease in the frequency of attacks at 3 months follow-up. The patient was seen by another physician who switched his treatment to dapsone and Imuran, however, the patient developed an allergic reaction to dapsone, so Imuran was continued alone with no improvement at 6 months, and colchicine was added to his treatment. The patient reported no clinical improvement with an increase in the frequency of the attacks that required days off from school. Consequently, he was started on intravenous tocilizumab every other week with corticosteroids. As ear-related symptoms did not improve, tocilizumab was discontinued and adalimumab 40 mg every other week was initiated. After adalimumab treatment, ear redness and pain improved but relapsed after 4 months.

**Figure 1:**
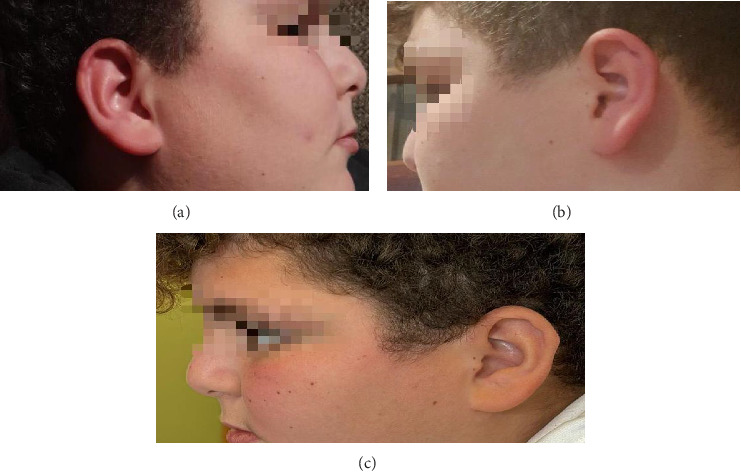
(a-b) Right and left-sided erythema of the ear. (c) At 1-year follow-up; no evidence of cartilage destruction or swelling.

Upon presentation, the patient was afebrile, and vital signs were within reference range. Physical examination was unremarkable except for unilateral auricular erythema associated with a burning sensation (Figure 1). A neurological examination did not reveal any anomalies. No change in the characteristics of his pain since its onset 5 years ago, except for daily attacks in the past 3 weeks, sufficiently painful that made him cry or awakened him from sleep. Occurrence of the attacks seems but not always to be triggered by stress, rubbing or touching the ear, heat, and relieved by ice packings. The patient denied arthralgia, epistaxis, headache, skin rashes. Initial investigation revealed a normal complete blood count, liver and kidney functions. C-reactive protein (CRP) was positive at 27 mg/L (reference range < 6 mg/L) and erythrocyte sedimentation rate (ESR) was elevated at 20 mm/first hour. Auto-immune work-up came back negative (Table 1). A brain and cervical spine magnetic resonance image (MRI) was negative. In light of these findings, the patient was diagnosed with idiopathic red ear syndrome (RES), and discharged on ibuprofen, a tricyclic antidepressant (TCA), and a calcium channel blocker (CCB). It was advised to keep a diary of episodes.

At the 1-month follow-up, the patient reported a decrease in the intensity of the attacks, while still experiencing a few attacks every other day. At a 2-month follow-up, the patient reported three attacks lasting 10 min in the past month. At 3-month, 6-month, and 1-year follow-ups, the patient reported no attacks (Figure 1).

## 3. Discussion

RES is an uncommon condition, particularly among the pediatric population. A review of 60 cases reported in the literature between 1996 and 2010, found 11 cases among the pediatric population, with seven patients experiencing bilateral attacks [2]. The most commonly reported triggers include touch, stress, heat, and lying on the affected side. Chewing, showering, neck movement, and cold exposure have also been reported as inducers of attacks. It is worth mentioning, that patients can experience spontaneous attacks. In a review published by Lambru, Miller, and Matharu 25 patients exhibited exclusively spontaneous attacks, while most had both spontaneous and triggered attacks [3].

While the pathogenesis of RES is not clearly understood, two forms of the disease have been identified; the primary or idiopathic form and the secondary form. Ten out of the 12 patients with RES originally described by Lance, had RES episodes thought to be caused by temporomandibular joint (TMJ) dysfunction, cervical arachnoiditis or spondylitis, and thalamic syndrome [4]. Primary RES can occur in isolation, and more recently, several cases also suggest its relationship to primary headache, most commonly with migraine, particularly among the pediatric population [5]. Raieli et al., described eight patients with migraine, who also suffered from RES, seven of whom were children and one adult, with RES attacks usually occurring in close temporal relation with migraine in all but one case [6]. In a study conducted with 226 children affected by primary headache, 76.4% were found to suffer from migraine, of which 23.3% also suffered from RES during migraine attacks [7]. These findings support the hypothesis of intrinsic dysregulation of the brainstem trigeminal autonomic circuit, rather than a local axonal reflex previously suggested by Lance.

Our pediatric patient was initially diagnosed with RP due to recurrent episodes of erythema and tenderness of the ear, lasting for hours. A thorough history from the patient revealed a partial resolution of the episodes followed by recurrent ones which would have explained the symptoms lasting for more than four to 6 hours, and sometimes according to the patient's parents for days. RP is an immune-mediated disease characterized by progressive destruction of the cartilaginous structures. Similar to RES, it is an uncommon condition, particularly among the pediatric population with most cases published as case reports and case series. Auricular chondritis is the most common presentation and can involve the outer, middle, and inner ears. It is classically characterized by swelling, erythema, and tenderness sparing the noncartilaginous lobule [8]. Children with RP usually present with arthritis, auricular chondritis, and respiratory tract chondritis. The involvement of major airways is more common and severe in children with RP compared to adults. Arthritis and nasal and auricular chondritis are the most common presentations in adults with RP [9]. A retrospective study from three French hospitals including 10 pediatric patients with RP and a mean follow-up duration of 14 years, revealed that auricular chondritis was the initial presentation in 80% of cases. All patients developed otologic disease within 5 years of disease onset. Nasal involvement was seen in 60% of cases, joint involvement in 90%, and laryngeal chondritis in 60%. Despite treatment, 90% had disease progression and destruction. The disease course was complicated by cardiac involvement in one adolescent that began initially with a complete atrioventricular block, followed by aortic regurgitation requiring aortic valve replacement which was further complicated by endocarditis and mitroaortic valve replacement [10]. Our pediatric patient, at the initial onset of the disease 5 years prior to presenting at our facility, and throughout the five year-period, denied any other symptoms related to cartilage involvement aside from recurrent episodes of erythema and tenderness of the ear, with attacks involving the ear lobules. Upon presentation, the physical examination revealed no deformity over the ear, nose, or skin lesions. The patient also denied headaches, epistaxis, cough, hoarseness, and joint pain. In light of these findings, RP was ruled out. Due to the association of secondary RES with upper cervical spine diseases and thalamic lesions, a cervical spine and brain MRI were done and yielded negative findings. Our patient was diagnosed with idiopathic RES further supported by the good response to treatment during follow-up.

RES is usually refractory to treatment. No specific treatment has been proven to be effective. Several drugs have been tried in cases in an open-label fashion. Most authors reported partial or marginal benefits at follow-up. Boulton et al. reported two cases treated with ibuprofen, leading to RES's resolution [11]. Flunarizine and nimodipine have been tried in patients with migraine-related RES, and treatment has led to a decrease in the frequency and intensity of the attacks [6]. In a series of 12 patients with RES, treated with gabapentin, eight patients reported a significant decline in the frequency of the attacks [12]. Our patient was treated with a combination of a nonsteroidal anti-inflammatory drug (NSAID), a CCB, and a TCA, with a significant reduction in attack frequencies and intensities and a complete resolution of attacks after 1 year.

## 4. Conclusion

We presented the case of a 14-year-old boy diagnosed with RES after being misdiagnosed with and treated for RP for five years. Pediatric RES is rare and its pathogenesis is poorly understood. While our patient benefited from a combination treatment of NSAIDs, CCBs, and TCAs, guidelines for optimal treatment are still lacking and more studies on this rare entity are needed.

## Figures and Tables

**Table 1:** Auto-immune work-up.

Investigation	Results	Normal values
ANA	1/100	Not available
SS-A native (SSA)	5	0–91 U/mL
SS-B (SSB)	3.1	0–73 U/mL
Scl-70 (Scl)	0	0–11 SI
Jo-1 (Jo)	11.3	< 40 AU/mL
Nucleosomes (NUC)	Negative	Not available
Histones (HI)	0.2	< 40 U/mL
Anti-ds DNA IgG (IU/mL)	10.9	0–29.9
Anti-Sm (U/mL)	3.2	0–7
ANCA-C (AU/mL)	2.8	≤ 19
ANCA-P (kU/L)	1.2	< 1.4
C3 (g/L)	1.2	0.9–1.8
C4 (g/L)	0.12	0.1–0.4
Angiotensin-converting enzyme (U/L)	44.7	16–85
Rheumatoid factor (U/mL)	6.54	< 20
Anticyclic citrullinated peptide (U/mL)	1.3	< 5

## Data Availability

The data that support the findings of this study are available on request from the corresponding author (G.E.K.). The data are not publicly available due to privacy or ethical restrictions.

## References

[1] Moitri M. O., Banglawala S. M., Archibald J. (2015). Red Ear Syndrome: Literature Review and a Pediatric Case Report. *International Journal of Pediatric Otorhinolaryngology*.

[2] Ryan S., Wakerley B. R., Davies P. (2013). Red Ear Syndrome: A Review of All Published Cases (1996–2010). *Cephalalgia*.

[3] Lambru G., Miller S., Matharu M. S. (2013). The Red Ear Syndrome. *The Journal of Headache and Pain*.

[4] Lance J. W. (1994). The Mystery of One Red Ear. *Clinical and Experimental Neurology*.

[5] Uca A. U., Kozak H. H. (2014). Coexistence of Migraine Headache and Red Ear Syndrome. *Noro Psikiyatr Ars*.

[6] Raieli V., Monastero R., Santangelo G., Eliseo G. L., Eliseo M., Camarda R. (2002). Red Ear Syndrome and Migraine: Report of Eight Cases. *Headache*.

[7] Raieli V., Compagno A., Brighina F. (2011). Prevalence of Red Ear Syndrome in Juvenile Primary Headaches. *Cephalalgia*.

[8] Alqanatish J. T., Alshanwani J. R. (2020). Relapsing Polychondritis in Children: A Review. *Modern Rheumatology*.

[9] Fonseca A. R., de Oliveira S. K., Rodrigues M. C., Aymoré I. L., Domingues R. C., Sztajnbok F. R. (2013). Relapsing Polychondritis in Childhood: Three Case Reports, Comparison With Adulthood Disease and Literature Review. *Rheumatology International*.

[10] Belot A., Duquesne A., Job-Deslandre C. (2010). Pediatric-onset Relapsing Polychondritis: Case Series and Systematic Review. *Jornal de Pediatria*.

[11] Boulton P., Purdy R. A., Bosch E. P., Dodick D. W. (2007). Primary and Secondary Red Ear Syndrome: Implications for Treatment. *Cephalalgia*.

[12] Al-Din A. S., Mir R., Davey R., Lily O., Ghaus N. (2005). Trigeminal Cephalgias and Facial Pain Syndromes Associated With Autonomic Dysfunction. *Cephalalgia*.

